# Metagenomic and Functional Characterization of Two Chilean Kefir Beverages Reveals a Dairy Beverage Containing Active Enzymes, Short-Chain Fatty Acids, Microbial β-Amyloids, and Bio-Film Inhibitors

**DOI:** 10.3390/foods11070900

**Published:** 2022-03-22

**Authors:** Claudia Ibacache-Quiroga, Karoll González-Pizarro, Mariam Charifeh, Christian Canales, Rodrigo Díaz-Viciedo, Oliver Schmachtenberg, M. Alejandro Dinamarca

**Affiliations:** 1 Escuela de Nutrición y Dietética, Facultad de Farmacia, Universidad de Valparaíso, Valparaíso 2360102, Chile; 2 Centro de Micro-Bioinnovación, Universidad de Valparaíso, Valparaíso 2360102, Chile; karoll.gonzalez@uv.cl (K.G.-P.); mariamcharifeh@gmail.com (M.C.); rodrigo.diaz@uv.cl (R.D.-V.); 3 Facultad de Ingeniería y Tecnología, Universidad San Sebastián, Concepción 4080871, Chile; christian.canales@uss.cl; 4 Escuela de Química y Farmacia, Facultad de Farmacia, Universidad de Valparaíso, Valparaíso 2360102, Chile; 5 Instituto de Biología, Facultad de Ciencias, Universidad de Valparaíso, Valparaíso 2360102, Chile; oliver.schmachtenberg@uv.cl; 6 Centro Interdisciplinario de Neurociencias (CINV), Universidad de Valparaíso, Valparaíso 2381850, Chile

**Keywords:** kefir, probiotic, curli, β-amyloids, biofilm inhibitor, short-chain fatty acids

## Abstract

Kefir beverage is a probiotic food associated with health benefits, containing probiotic microorganisms and biomolecules produced during fermentation. The microbial composition of these beverages varies among countries, geographical regions, and the substrates, therefore, the characterization of kefir beverages is of great relevance in understanding their potential health-promoting and biotechnological applications. Therefore, this study presents the metagenomic and functional characterization of two Chilean kefir beverages, K02 and K03, through shotgun and amplicon-based metagenomic, microbiological, chemical, and biochemical studies. Results show that both beverages’ microbiota were mainly formed by Bacteria (>98%), while Eukarya represented less than 2%. Regarding Bacteria, the most abundant genera were *Acetobacter* (93.43% in K02 and 80.99% in K03) and *Lactobacillus* (5.72% in K02 and 16.75% in K03), while *Kazachstania* was the most abundant genus from Eukarya (42.55% and 36.08% in K02 and K03). Metagenomic analyses revealed metabolic pathways for lactose and casein assimilation, biosynthesis of health-promoting biomolecules, and clusters for antibiotic resistance, quorum sensing communication, and biofilm formation. Enzymatic activities, microbial β-amyloids, and short-chain fatty acids (acetic acid and propionic acid) were also detected in these beverages. Likewise, both kefir beverages inhibited biofilm formation of the opportunistic pathogen *Pseudomonas aeruginosa*.

## 1. Introduction

Kefir is an ancestral dairy beverage originating from the Russian Caucasus and the Tibetan regions, produced by a microbial consortium through fermentation. This microbial consortium is composed of bacteria and yeast, naturally packed into a macrostructure known as “kefir grains” [1] that is used as the starter for the fermentation process. The microbial composition of kefir varies among countries, geographical regions, and due to the substrates used for fermentation [2,3,4]; therefore, different bacterial and yeast genera have been identified in kefir beverages and kefir grains [1,2,3]. Among commonly isolated bacterial genera are *Lactobacillus*, *Lactococcus*, *Luconostoc*, *Acetobacter*, *Enterococcus*, *Pediococcus*, and *Gluconobacter* [4,5,6,7,8], while the yeast component includes *Saccharomyces*, *Candida*, *Kluyveromyces*, *Torulaspora*, *Kazachstania*, *Rhodosporidium*, *Pichia*, and *Yarrowia* [9,10,11].

In the kefir grains, the microorganisms are embedded within an extracellular matrix composed of proteins and polysaccharides [12], forming a microbial ecosystem where microbe-microbe, microbe-environment, and microbe-substrate interactions occur [13,14]. The microorganisms of this ecosystem metabolize fat, proteins, and carbohydrates present in milk, generating different high-value biomolecules, such as vitamins and organic compounds, with known health benefits [15,16].

The potentially beneficial properties of kefir beyond its nutritional value have aroused scientific interest, and diverse studies have explored its effects on human health. At the intestinal level, kefir modulates the gut microbiota [17,18,19], reducing the Firmicutes/Bacteroidetes ratio and inhibiting the proliferation of opportunistic bacteria from the *Enterobacteriaceae* family [19]. Likewise, bacterial strains isolated from kefir exert a protective effect against gastrointestinal pathogens [20,21]. Furthermore, kefir consumption induces enzymatic activity within the small intestine, increasing protein metabolism, glucose absorption, and lactose hydrolysis, reducing the physiologic and symptomatic parameters associated with lactose intolerance [22,23,24]. This dairy beverage also improves non-alcoholic fatty liver syndrome [25,26], and its cell-free fraction induces apoptosis of gastric and colorectal cancer cells [27,28]. Outside the gastrointestinal tract, kefir has shown an anti-inflammatory effect [29], wound-healing properties [30], as well as the ability to improve atopic dermatitis [31].

The beneficial effects of kefir beverages are tightly related to their microbial composition and productive conditions. In this context, during the past decade, the development and massification of new generation sequencing (NGS) have allowed the identification of the microbial structure in kefir beverages and other fermented foods and the metabolic genetic networks of the microbial community through amplicon-based [32,33,34,35,36,37] and shotgun metagenomic sequencing [38,39,40,41,42], respectively. Even though most metagenomic studies have focused on the kefir grain microbiota [32,33,36,37,38,41,43], during the past years the analysis of the kefir beverages has gained relevance [35,39,42,44,45].

The present work studied two Chilean kefir beverages, produced by two different kefir grains of household origin, collected from the Valparaíso region and selected due to their different physical (macroscopic) characteristics. The Chilean kefir beverages were analyzed at the chemical, amplicon-based and shotgun metagenomic, nutritional, and microbiological levels with an emphasis on their microbiota’s structure and function, their enzymatic activities, and the presence of biomolecules that may contribute to their health-promoting benefits through modulation of the gut microbiota and concomitantly the gut-brain axis, and the inhibition of potentially pathogenic bacteria. To our understanding, this is the first multi-analyses study of kefir beverages of Chilean origin.

## 2. Materials and Methods

### 2.1. Materials

The kefir grains used in this study for the production of K02 and K03 beverages belong to the microbial collection of the Centro de Micro-Bioinnovación, of the Universidad de Valparaíso. Both kefir grains are of household origin and were collected in the Valparaíso Region (Chile). Commercial UHT-treated raw milk (3.1% *w*/*v* fat) was used for kefir preparation. For proximate analysis, petroleum ether, copper (III) sulfate, potassium sulfate, sulfuric acid, ethanol, sodium hydroxide, and boric acid were purchased from Merck KGaA (Darmstadt, Germany), and Total Dietary Fiber Assay Kit, Methyl Red, and Methyl Blue were purchased from Sigma-Aldrich, Co. (St. Louis, MO, USA). All antibiotics and short-chain fatty acids standards were from Sigma-Aldrich (St. Louis, MO, USA). For microbial growth, MRS and Sabouraud agar were purchased from Sigma-Aldrich, Co. (St. Louis, MO, USA) and Merck KGaA (Darmstadt, Germany), respectively. Marine broth 2216, MacConkey agar, tryptone, yeast extract, casaminoacids and agar were from Becton, Dickinson and Co. (Sparks, NV, USA), D-glucose and skim milk powder from Sigma-Aldrich, and sodium chloride from Loba Chemie Pvt. Ltd. (Mumbai, India). For curli analysis, Congo Red and Coomassie-Brilliant Blue G-250 were purchased from Merck KGaA (Darmstadt, Germany), while curcumin from Sigma-Aldrich, Co. (St. Louis, MO, USA). For biofilm formation assays, crystal violet was obtained from Merck KGaA (Darmstadt, Germany). For metagenomic assays, Meta-G-Nome DNA Isolation Kit (Epicentre) was obtained from Epicentre (Madison, WI, USA) and TruSeq Nano DNA LT Kit from Illumina Inc. (San Diego, CA, USA). For biochemical characterization, API 20E system was purchased from bioMérieux (Lyon, France).

### 2.2. Culture Media

The composition of non-commercial culture media is detailed in Supplementary Table S1. When needed, antibiotics were added to culture media at the following concentrations: ampicillin (AMP) at 100 µg/mL, chloramphenicol (CLP) at 50 µg/mL, fosfomycin (FOS) at 50 µg/mL, and kanamycin (KAN) at 50 µg/mL.

### 2.3. Production of Kefir Beverage and Whey

To produce kefir beverages, two different kefir grains were used as starters. These grains were selected due to their macroscopic differences (Supplementary Figure S1). Kefir beverages were produced by mixing 10 g of kefir grains with 100 mL of UHT-treated raw bovine milk and incubating at 25 °C for 48 h. After fermentation, the kefir beverage was separated from the kefir grains by percolation. Kefir whey was obtained from kefir beverages by centrifugation at 8000 rpm at 10 °C for 30 min. To obtain cell-free kefir whey, supernatants were extracted and filtered through a 0.22 µm sterile membrane.

### 2.4. Characterization of Kefir Beverages

#### 2.4.1. Nutritional Properties

pH was determined using a portable pHmeter (EZDO, PP-203, Taipei City, Taiwan) at 25 °C, in triplicates. Nutritional properties of kefir were determined by proximate analysis according to AOAC (2006) [46]. Briefly, total ash was determined by weighing 2 g of each kefir beverage into an ashing dish and incinerating them at 650 °C for 8 h. After incineration, samples were cooled down in a desiccator and weighed when they reached room temperature. For moisture content calculation, ceramic capsules were dehydrated at 105 °C, chilled to room temperature, and weighed. Then, 1 g of each sample was placed into a capsule and dried at 105 °C until a constant weight was achieved. The moisture content of kefir beverages was estimated by the weight loss of the samples. The protein content in kefir beverage was determined by measuring nitrogen content from 1 g of sample, using the Kjeldahl method, and multiplying it by 6.25. For the determination of lipid content, total lipids were extracted from 2 g of kefir beverages with petroleum ether in a Soxhelt apparatus at 70 °C for 4 h. The ether extracts were placed into a pre-weighed Erlenmeyer flask, the solvent was evaporated, and the lipid content was calculated by measuring the weight difference of the flasks. The percentage of carbohydrates in each sample was estimated as the following formula: % Carbohydrates = 100 − % ash −% moisture − % proteins − % lipids. The total calories were calculated by multiplying the content of lipids, proteins, and carbohydrates by their caloric contribution: 9 kcal/g for lipids and 4 kcal/g for proteins and carbohydrates. Total dietary fiber was determined using the enzymatic-gravimetric method. For this, kefir beverages were pretreated with petroleum ether to remove fat. The obtained residue was enzymatically digested and precipitated using the Total Dietary Fiber Assay Kit (Sigma-Aldrich), according to the manufacturer’s instruction. To estimate the amount of crude fiber, ash and protein were determined in the obtained precipitates, and it was calculated as follows: Total dietary fiber (%) = 100 − % ash − % protein. All samples were analyzed in triplicates.

#### 2.4.2. Detection of Short-Chain Fatty Acids

Detection and quantification of short-chain fatty acids (SCFA) in kefir beverages was performed through HPLC as described by Asarat and coworkers based on Donkor’s protocol [47,48]. The SCFA evaluated in this study were acetic acid, propionic acid, and butyric acid. For SCFA quantification, standard curves of the mentioned SCFA were generated using solutions of predetermined concentrations.

#### 2.4.3. Microbial Viability in Kefir Beverages

Microbial viability in kefir beverage was evaluated by colony-forming unit (CFU) counts. For CFU counts, serial dilutions of kefir beverage in NaCl 0.9% *w*/*v* were performed. A total of 100 µL of each dilution were plated on skim milk agar, MRS agar, GYC agar, and Sabouraud agar, in triplicates, for microbial, lactic acid bacteria, acetic acid bacteria, and yeast isolation, respectively. Plates were incubated at 26 °C for 48 h. For lactic acid bacteria quantification, cultures were incubated at 37 °C using anaerobic bags with a CO_2_ atmosphere. To evaluate the presence of gram-negative enteric bacteria, samples were plated on MacConkey agar and incubated at 37 °C for 48 h. Additionally, bacterial susceptibility to ampicillin, chloramphenicol, fosfomycin, and kanamycin, was evaluated on skim milk agar, MRS agar, and GYC agar under the previously described conditions.

### 2.5. Metagenomic Sequencing

#### 2.5.1. DNA Extraction

For metagenomic sequencing, total DNA from each kefir beverage was extracted using Meta-G-Nome DNA Isolation Kit (Epicentre) according to the manufacturer’s instructions. DNA integrity was confirmed by agarose electrophoresis, and DNA concentration was determined by UV absorbance using a QuantiFluor^®^ fluorometer (Promega, Madison, WI, USA).

#### 2.5.2. Shotgun Metagenomic Sequencing and Analysis of Kefir Beverages

Total DNA libraries were generated from 100 ng of metagenomic DNA using TruSeq Nano DNA LT Kit (Illumina). Quality control of the genetic libraries was performed using a 2100 Bioanalyzer (Agilent Technologies, Santa Clara, CA, USA). Metagenomic DNA was sequenced through shotgun metagenomic DNA sequencing in an Illumina HiSeq4000 system using a pair-end protocol (2 × 250 bp). Sequence quality was analyzed using the Prinseq tool (v. 0.20.4) and Fasta QC (v. 0.11.5) [49]. Low-quality sequences at left and right extremes and Illumina-adaptor sequences were trimmed off. Sequences with Phred values < 33, with more than a 10% of ambiguous bases or with a total length below 100 bp were not considered for further analyses. Metagenomic analyses included the following key steps: identification of 16S rRNA codifying sequences, operational taxonomic unit (OTU) generation, metagenome phylotyping, sequence assembly, open reading frame (ORF) identification and clustering, and functional annotation of clustered ORFs. Taxonomic profiles of kefir samples were determined using MetaPhlan2 software (v. 2.0) [50]. For the alignment and taxonomic profiling of high-quality reads, Bowtie2 software was used as reference [51]. For gene identification and functional annotation, de novo assembly of high-quality reads was carried out using MetaSPAdes software (v. 3.11.0) [52]. Gene prediction was performed using MetaGeneMark software (v. 3.26) [53], while functional annotation of assembled sequences was done using eggNOG-Mapper software (v. 1.0.1) [54]. Alignment of the predicted proteins was performed using BLASTp (v. 2.6.0), with the Swissprot database as reference. Additionally, predicted genes were annotated using DIAMOND software (v. 0.9.10) [55], GhostKOALA [56] and MG-RAST (v. 4.0.3) [57].

#### 2.5.3. Amplicon-Based 16S rDNA Metagenomic Sequencing

For 16S rDNA analysis, the V4 variable region of 16S rDNA was amplified from 10 ng of total DNA from each sample through qPCR using the protocol developed by Caporaso and coworkers [58]. Length, integrity, and distribution of 16S rDNA metagenomic libraries were estimated using the 2100 Bioanalyzer (Agilent Technologies), and 16S rDNA libraries were sequenced using an Illumina MiSeq platform with a 2 × 150 bp protocol. Pair-end sequences were filtered according to quality control parameters using PRINSEQ software (v. 20.4) [49]. Illumina adapter sequences and sequences with a Phred score of ≤30 were trimmed off, and quality-filtered sequences were merged. High-quality sequences were analyzed using Quantitative Insights into Microbial Ecology (QIIME, v1.9) [59]. To determine relative microbial abundance in kefir beverages, OTUs were identified and analyzed. OTUs were generated using the SILVA Database (v. 132) as reference. Non-assigned sequences were clustered against each other using de novo clustering. In both cases, clustering was performed using UCLUST [60], considering 97% of identity. Each OTU is formed by at least five sequences, and one sequence per cluster was selected for further analysis. Chimeric sequences were identified and eliminated using the ChimeraSlayer method. Results were analyzed using MEGAN (v. 6) and MG-RAST software (v. 4.0.3) [57,61].

### 2.6. Enzymatic Activity in Kefir Beverages

Enzymatic activity of kefir beverages and kefir whey was evaluated using the API 20E System (bioMérieux) according to the manufacturer’s instructions. Briefly, 100 µL of each kefir beverage were resuspended in 7 mL NaCl solution (0.9% *w*/*v*), mixed through vortexing, and inoculated into API 20E strips. For enzymatic activity of kefir whey, cell-free whey was directly inoculated into API 20E strips. In both cases, strips were incubated at 25 °C for 48 h. Casein hydrolysis of kefir beverages and whey was evaluated by plating 100 µL of each sample into a standardized hole (8 mm) in a skim milk agar plate (30 mL). Plates were incubated at 25 °C and casein hydrolysis was measured after two, four, and seven days. Casein hydrolysis was observed as the clearance halo around the inoculation spot and expressed in millimeters (mm). The caseinase activity of all samples was evaluated in triplicates.

### 2.7. Effect of Kefir Beverages on Biofilm Formation

The effect of kefir beverages on biofilm formation was carried out using three kefir-derived products: (i) cell-free kefir suspension; (ii) cell-free kefir whey; and (iii) denaturized kefir whey. For cell-free kefir suspension, kefir beverage was diluted in NaCl 0.9% *w*/*v* in a 1:1 ratio and filtered through a 0.22 µm sterile membrane, while kefir whey was produced as detailed in Section 2.3. Denaturized kefir whey was obtained by incubating kefir whey at 95 °C for 10 min. The effect of these samples on biofilm formation was tested by the crystal violet method according to O’Toole and coworkers on 96-well plates [62], using two bacterial models: the non-pathogenic marine bacterium *Cobetia marina* (ATCC 25374); and the opportunistic bacterium *Pseudomonas aeruginosa* PAO1. For this purpose, *C. marina* was grown in Marine Broth 2216 at 26 °C for 48 h, while *P. aeruginosa* in LB broth at 37 °C for 24 h.

### 2.8. Detection of β-Amyloids in Kefir Beverage

For detection of β-amyloid proteins in kefir beverage, samples were plated on YESCA agar plates supplemented with Congo Red (CR) (50 µg/mL) and Coomassie Brilliant Blue G-250 (1 µg/mL). CR can stain β-amyloid and cellulose, therefore, specific detection of β-amyloid proteins was conducted on curcumin (18 µg/mL)-supplemented YESCA plates [63]. Detection of cellulose in kefir beverages was performed on YESCA agar supplemented with calcofluor (1µg/mL) [64]. *Escherichia coli* strain BW25113 and its Δ*csgA*::Km derivative KW1025 [65] were used as positive and negative controls for curli production, a β-amyloid protein, respectively. Plates were incubated at 25 °C for 48 h, and the production of β-amyloid proteins and cellulose was determined.

### 2.9. Statistical Analysis

Statistical analysis was carried out using StatPlus:mac LE (Analysoft Inc.). For all analyses, two-sample, paired *t*-tests were performed, and the level of significance was set at 95% (*p* < 0.05).

### 2.10. Accession Numbers

Sequence data reported in this work have been submitted to the National Center for Biotechnology Information (BioProject PRJNA388572). K02 and K03 metagenomes were submitted as BioSamples under accession numbers SAMN09389955 and SAMN08943003, respectively. Raw sequence data of shotgun metagenomic sequencing were deposited under accession SRR8282406 (K02) and SRR7287342 (K03). Raw sequencing data of 16S rDNA sequencing was deposited under the following accession numbers: SRR10232092 (K02) and SRR10233296 (K03).

## 3. Results

### 3.1. Nutritional and Microbiological Characterization of Kefir Beverages

The kefir beverages analyzed in this study, K02 and K03, are acidic products with pH of 4.3. Their nutritional composition showed that the most abundant components were lipids (3.41% *w*/*v* in K02 and 3.15% *w*/*v* in K03) and proteins (3.06% *w*/*v* in K02 and 3.07% *w*/*v* in K03) (Table 1). K02 presented a higher lipid content compared to K03 (*p* < 0.05), while K03 showed a higher concentration of carbohydrates (*p* < 0.05) than K02. No significant differences were observed in protein and dietary fiber content between both dairy beverages (*p* > 0.05). Regarding short-chain fatty acids, K02 and K03 contained lactic acid, acetic acid, and propionic acid, where lactic acid was the most abundant in both samples (Table 1). Significant differences between the two beverages were only observed in acetic acid with a higher content in K02 (*p* < 0.05).

Total viable culturable microorganisms in kefir beverages reached 3 × 10^9^ UFC/mL, with acetic acid bacteria being the most abundant in both samples (2 × 10^9^ UFC/mL in K02 and 3 × 10^9^ UFC/mL in K03), followed by yeasts (3 × 10^8^ UFC/mL in K02 and 2 × 10^8^ UFC/mL) and lactic acid bacteria (1 × 10^8^ UFC/mL both beverages) (Table 1). No colony-forming units of Enterobacteria were detected on MacConkey agar in either sample.

### 3.2. Taxonomic Analysis of Kefir Beverages

**Figure 1:**
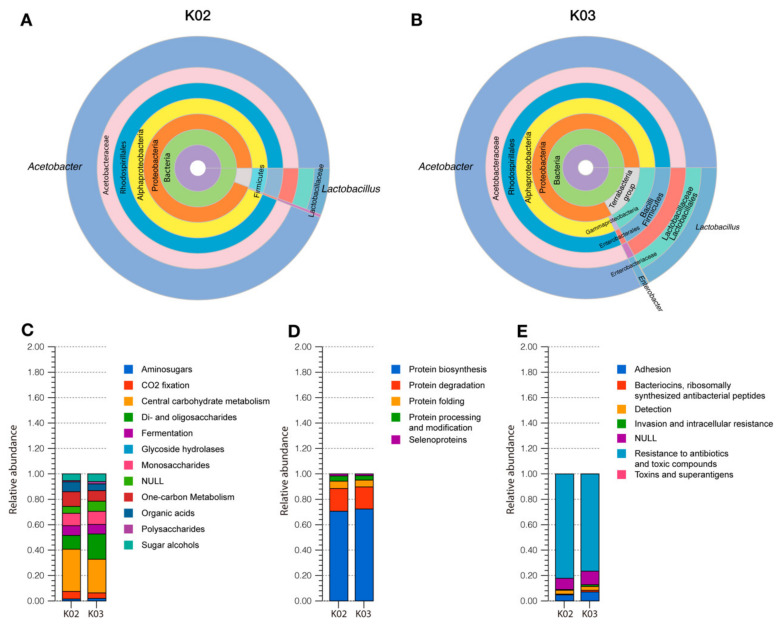
Taxonomic and metagenomic features of kefir beverages. Bacterial taxonomic analysis of the metagenomes of kefir beverages through 16S rDNA sequencing, represented as bacterial relative abundance (**A**,**B**). Subsystem analysis of kefir beverages metagenomes: *Carbohydrates* (**C**), *Protein metabolism* (**D**), and *Virulence, Disease, and Defense* (**E**).

The analysis of operational taxonomic units (OTUs) from shotgun metagenomic sequencing showed that Bacteria was the most abundant domain with 98.82% and 99.65% in K02 and K03, respectively, while Eukarya represented a 1.11% (K02) and a 0.25% (K03). The taxonomic analysis of the Eukarya domain revealed that *Kazachstania*, *Saccharomyces*, and *Citeromyces* were the most abundant genera. Bacterial taxonomic analysis, performed through amplicon-based sequencing and analysis of the 16S rDNA (Statistics of the assembly are detailed in Supplementary Table S2), indicated that the main genera in kefir beverages were *Acetobacter* (93.43% in K02 and 80.99% in K03) and *Lactobacillus* (5.72% in K02 and 16.75% in K03) (Figure 1A,B). OTUs of bacteria belonging to the *Enterobacteriaceae* family were also detected; however, the relative abundances of these microorganisms were lower than 2.2% (0.37% in K02 and 2.18% in K03).

### 3.3. Functional Characterization of Kefir Beverages

The functional characterization of kefir beverages was performed by two different approaches: metagenomic analyses and confirmation of the functionality of these genes through chemical, biochemical and microbiological assays.

For shotgun metagenomic sequencing, a total of 24,349 and 33,094 genes were predicted in K02 and K03, respectively, and the statistics of the analysis are detailed in Supplementary Table S3.

#### 3.3.1. Carbohydrate Metabolism

According to functional annotation using subsystem analysis of K02 and K03 metagenomes, carbohydrates represented about 12% in both samples (Supplementary Figure S2). From this, the genes involved in di- and oligosaccharide metabolism represented 10.8% in K02 and 19.8% in K03 (Figure 1C, Supplementary Figure S3A), where maltose, maltodextrin, lactose, and β-glucosides metabolism were the most abundant in both samples (Supplementary Figure S3B). Genes encoding β-galactosidase enzymes, *lacZ*, and *lacG*, were identified in both kefir samples through KEGG Orthology-based analysis and were attributed to the class Bacilli. Monosaccharide metabolism represented 9.7% and 10.3% of the carbohydrate subsystem in K02 and K03 metagenomes, respectively (Figure 1C, Supplementary Figure S3C). Fermentative metabolism was also identified through subsystem analysis, being acetoin and butanediol metabolism, lactic fermentation, and mixed acid fermentation the most abundant within this group of genes (Supplementary Figure S3D). Regarding lactic fermentation, *ldh* and *ldhA,* encoding L-lactate and D-lactate dehydrogenases, responsible for the production of L-lactate and D-lactate from pyruvate, respectively, were identified in both beverages. D-lactate biosynthesis was associated with bacteria belonging to Firmicutes, Alphaproteobacteria, Gammaproteobacteria, and Ascomycetes, while genes involved in L-lactate biosynthesis were attributed to Firmicutes.

Related to mixed acid fermentation, the complete genetic pathways to produce acetate and propanoate, two short-chain fatty acids (SCFA), were identified. Chemical analyses of the kefir beverages revealed that both samples contained acetic acid and propionic acid produced by glucose fermentation, while butyric acid was not detected (Table 1). Likewise, the evaluation of the enzymatic activity of kefir beverages revealed that K02 and K03 presented β-galactosidase activity and anaerobically metabolized glucose to produce acetoin (Table 2). On the other hand, kefir whey of K02 (K02W) and K03 (K03W) metabolized glucose, mannitol, inositol, sorbitol, rhamnose, sucrose, melibiose, amygdalin, and arabinose.

#### 3.3.2. Protein Metabolism

**Figure 2:**
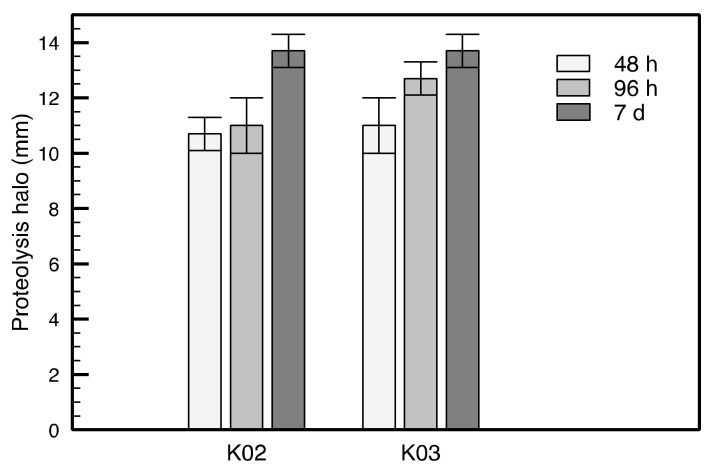
Casein hydrolysis activity of kefir beverages. Hydrolysis of casein was evaluated measuring proteolysis halo on skim milk agar. Caseinase activity was measured in triplicates and error bars represent standard deviation.

Protein metabolism was the third most abundant subsystem in kefir beverages metagenomes (Figure 1D, Supplementary Figure S4A), where biosynthesis (70%) and degradation (17%) were the main subgroups (Supplementary Figure S4B). Genes belonging to the degradation of proteins included caseinolytic proteases (ClpPs) in both samples (Supplementary Figure S4C) and were mainly attributed to Bacilli and Alphaproteobacteria. The evaluation of the caseinase activity on modified skim milk agar confirmed the proteolytic activity of both beverages (Figure 2). No significant differences between both kefir beverages (*p* > 0.05) were observed in this parameter at all the evaluated times.

#### 3.3.3. Microbial Resistance to Antimicrobials: Heavy Metals and Antibiotics

In K02 and K03, the Virulence, Disease, and Defense subsystem represented 3.28% and 2.91%, respectively (Supplementary Figure S2), in which antibiotic and toxin resistance genes were the most abundant (83.73% in K02 and 83.63% in K03) (Figure 1E, Supplementary Figure S5A). Regarding antibiotic resistance, multidrug resistance efflux pumps were the most abundant subgroup with 28.14% in K02 and 20.51% in K03 (Supplementary Figure S5B). Other genetic markers that confer resistance to β-lactams, fluoroquinolones, erythromycin, and fosfomycin were also detected on kefir metagenomes.

**Figure 3:**
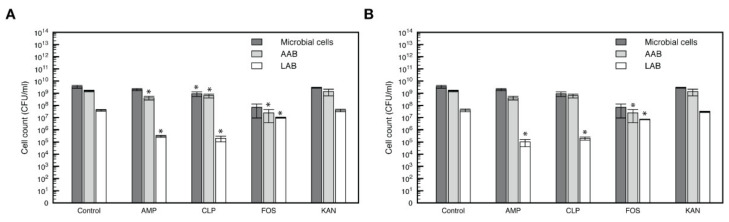
Bacterial susceptibility to antibiotics in kefir beverages. Count of total microbial cell, acetic acid bacteria (AAB) and lactic acid bacteria (LAB) in kefir beverages K02 (**A**) and K03 (**B**) supplemented with ampicillin (AMP) at 100 μg/mL, chloramphenicol (CLP) at 50 μg/mL, fosfomycin (FOS) at 50 μg/mL, and kanamycin (KAN) at 50 μg/mL. Kefir beverages produced in absence of antibiotics were used as controls. All microbial counts were evaluated in triplicates and error bars correspond to standard deviations. Asterisks denote statistically significant changes (*p* < 0.05).

To evaluate bacterial resistance to antibiotics in kefir beverages, the susceptibility of these microorganisms to ampicillin, chloramphenicol, fosfomycin, and kanamycin was determined. In K02, the microbial count on skim milk agar was significatively reduced in the presence of chloramphenicol and fosfomycin (*p* < 0.05) (Figure 3A). On the other hand, ampicillin, chloramphenicol, and fosfomycin significantly reduced acetic acid bacteria and lactic acid bacteria counts (*p* < 0.05) in this beverage. In K03, no significant changes in the microbial count were produced in the presence of the evaluated antibiotics (*p* > 0.05) on skim milk agar, while a decreased bacterial count of acetic acid microorganisms was observed on GYC agar supplemented with fosfomycin (*p* < 0.05) (Figure 3B). Additionally, lactic acid bacteria counts were decreased on MRS agar supplemented with ampicillin, chloramphenicol, and fosfomycin.

#### 3.3.4. Microbial Interaction in Kefir Beverages: Biofilm Formation and Host Interaction

**Figure 4:**
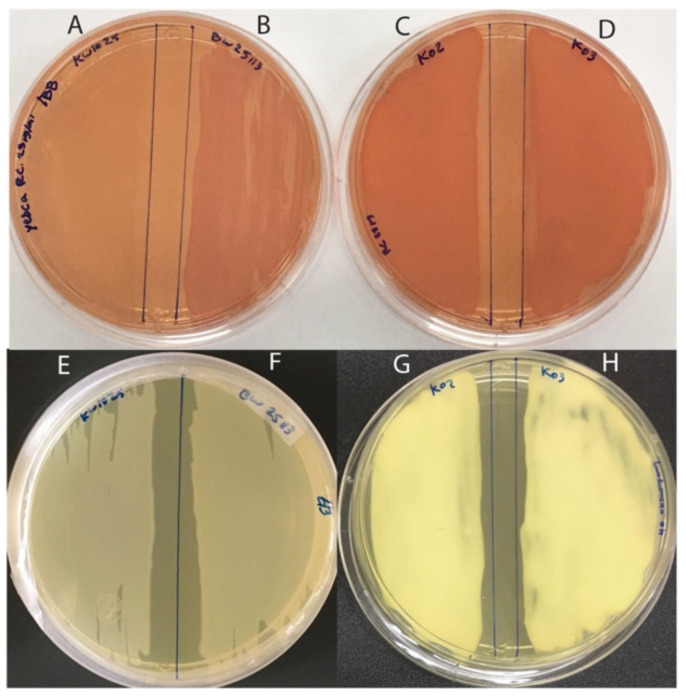
Detection of β-amyloid protein curli in kefir beverages. Presence of curli in kefir beverages K02 (**C**,**G**) and K03 (**D**,**H**) evaluated on YESCA agar supplemented with Congo red (25 µg/mL) and Coumassie-Brilliant Blue G-250 at (1 µg/mL) (**A**–**D**) and YESCA agar supplemented with curcumin (18 µg/mL) (**E**–**H**). *Escherichia coli* strain KW1025 (Δ*csgA*::Km) was used as negative control (**A**,**E**) and *E. coli* strain BW25113 was used as positive control for amyloid protein (curli) production (**B**,**F**).

Bioinformatic analyses of K02 and K03 metagenomes revealed the presence of genes involved in biofilm initiation and biofilm dispersal. Regarding biofilm initiation, the *csgBA* and *csgDEFG* operons responsible for the formation of curli, a β-amyloid protein, were detected. The presence of curli in kefir beverages was tested with Congo Red (CR) and curcumin staining. The results showed that K02 and K03 were labeled when plated on CR agar (Figure 4C,D). CR can stain other bacterial products, therefore, complementary assays using curcumin staining and a calcofluor-supplemented agar were used to detect curli and cellulose, specifically, confirming that K02 and K03 contained curli (Figure 4G,H) and that cellulose is not produced in kefir beverages (Supplementary Figure S6).

**Figure 5:**
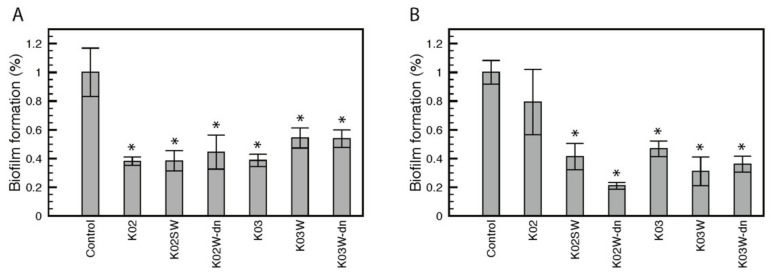
Effect of kefir on biofilm formation. The effect of kefir beverages (K02 and K03), kefir whey (K02W and K03W), and denaturized kefir whey (K02W-dn and K03W-dn) on biofilm formation was evaluated for *Pseudomonas aeruginosa* (**A**) and *Cobetia marina* (**B**). Results are expressed as biofilm formation ratio compared to the control condition. Samples were evaluated in triplicates and error bars represent standard deviation. Asterisks denote statistically significant changes (*p* < 0.05).

Related to biofilm regulation, the following genes were identified in this study: biofilm regulators *bssR* and *bssS*, toxin-antitoxin biofilm protein *tabA*, biofilm growth-associated repressor *bigR*, and biofilm dispersal mediator *bdcA*. In this context, the effect of kefir beverages on biofilm formation of opportunistic and environmental bacteria *Pseudomonas aeruginosa* PAO1 and *Cobetia marina*, respectively, was evaluated using cell-free kefir (K02 and K03), kefir whey (K02W and K03W), and denaturized kefir whey (K02W-dn and K03W-dn). The results demonstrate a 60% decrease in biofilm formation of *P. aeruginosa* in the presence of K02, K03, K02W and K02W-dn, while K03W and K03-dn reduced biofilm formation by 50% approximately (Figure 5A). On the other hand, *C. marina* biofilm formation was significantly reduced in the presence of all samples excluding K02. The highest effect was observed with K02W-dn with a reduction close to 80% (Figure 5B). No significant differences (*p* > 0.05) in biofilm formation of *P. aeruginosa* and *C. marina* were observed between K02 and K03. Differences between K02W and K03W were only observed in *P. aeruginosa*, while in *C. marina*, denaturized whey from both kefir beverages showed significant differences (*p* < 0.05). In both cases, K02 derived whey (K02W and K02W-dn) showed the highest biofilm inhibition.

## 4. Discussion

The microbial composition of kefir varies among different geographical regions, substrates, and fermentation conditions, with lactic acid bacteria being the most common type of microorganism in this dairy product. In this study, the taxonomic analysis of these beverages’ microbiota revealed that the dominant genus is *Acetobacter*. In both kefir samples, these acetic acid bacteria (AAB) represent more than 80% of the bacterial community, with K02 showing the higher proportion (93.43%). *Acetobacter* has been identified in different fermented foods like kombucha [66], cocoa beans [67], palm wine [68], and kefir [69]. In contrast to lactic acid bacteria (LAB), that have been identified in all kefir beverages, the presence of acetic acid bacteria in these dairy beverages is highly variable, with relative abundance that ranges from 0% to 90% [3,4,39]. Despite this variability, currently, in most metagenomically characterized kefir beverages, this genus represents less than 50% of the total bacterial community. For example, Marsh and coworkers characterized the microbiota of 15 kefir beverages from different countries, finding that in 67% of these samples, *Acetobacter* corresponded to 50% or less, with 37% kefir samples containing no AAB [3]. A factor that could explain the higher abundance of *Acetobacter* in K02 and K03, compared to other kefir beverages, is related to the production process of this study, specifically regarding the incubation period. During the fermentation, a successional phenomenon occurs where the AAB fraction tends to increase over time [44]. In this context, our 48-h fermentation process differs from other studies with 12–24 h incubation periods and could be influencing the microbial content of kefir beverages. Additionally, the difference in the microbiota composition might be related to the geographical origin of the kefir grains used as starters for fermentation. Nevertheless, since there are no previous studies of Chilean kefir beverages at a metagenomic level, further studies focused on the microbiota and the dynamics of this microbial ecosystem during fermentation in Chilean kefir beverages are needed.

With less than 20%, LAB species represented a minor component of these Chilean kefir beverages, which differs from previous studies where LAB from the phylum Firmicutes were the most abundant microorganisms in kefir beverages [9,21,33,34,35,45,70]. Among lactic acid bacteria, the only genus identified in K02 and K03 was *Lactobacillus.* This result differs from previous studies showing that LAB in kefir comprise different bacterial genera, including *Lactococcus*, *Lactobacillus*, and *Leuconostoc* [3,34,40].

A minor proportion of the kefir beverages microbiota was represented by yeast belonging to the *Saccharomycetaceae* family. The genera *Kazachstania* and *Saccharomyces,* which have been previously identified in kefir beverages and grains [71,72,73], were the most abundant in both samples. Despite its low abundance in kefir beverages, it is important to highlight that the metagenomic analyses of this study showed that *Saccharomycetaceae* contributed to the complete biosynthetic pathway of B-complex vitamins and ergosterol, genetic pathways for protein hydrolysis, and alcoholic fermentation of glucose. These metabolic pathways of the yeast present in the Chilean beverages are known to contribute to their organoleptic and antimicrobial properties [74,75].

Kefir is considered a health-promoting food due to its content of probiotic microorganisms. In our study, the main microbial component of the kefir beverages microbiota was bacteria from the genus *Acetobacter*. Despite not being a classic probiotic genus, probiotic species of *Acetobacter* have been isolated from dairy products [76,77]. On the other hand, the genus *Lactobacillus*, the second most abundant in the studied, are naturally part of different human microbiotas, and several species/strains are considered probiotic microorganisms [78,79,80,81,82]. The yeast component of these beverages included *Saccharomyces* and *Kazachstania*, two genera with proven probiotic features [72,83,84,85,86]. Despite these promissory results, further strain-specific analyses of isolated bacteria and yeast are needed to confirm their probiotic features. A traditional application of probiotics is the treatment of dysbiosis induced by antibiotics; nevertheless, antimicrobial therapy can affect their viability. In this context, antibiotic resistance observed in AAB and LAB in Chilean kefir may represent an advantage for the concomitant treatment with antibiotics.

Metabolic activity in kefir beverages is also of great relevance for its beneficial effects on human health. One of the primary nutrients in bovine milk is lactose, a disaccharide formed by glucose and galactose. In kefir, lactose is hydrolyzed by the microbial community through the enzymatic activity of β-galactosidase, reducing its concentration and producing glucose and galactose, which are used to form kefiran, the main exopolysaccharide in kefir [87]. In this context, consumption of kefir has been shown to improve symptoms of lactose intolerance [24], a worldwide syndrome that affects about 70% of the adult population, which is caused by a decreased production of lactase in the small intestine [88]. In the present study, genes involved in lactose metabolism and absorption were detected, while the functionality of these genes was confirmed through enzymatic assays (Supplementary Table S3) in K02 and K03. In addition to lactose metabolism, the production of short-chain fatty acids (SCFA) was detected in kefir beverages. These compounds are usually produced in the colon by microorganisms of the gut microbiota and are used as an energy source by enterocytes. They regulate intestinal motility and have an anti-inflammatory effect [89]. Quantification of SCFA in K02 and K03 showed that acetic acid was the most abundant SCFA, followed by propionic acid (Table 1). These results agree with metagenomic studies that identified the complete genomic pathways for synthetizing these SCFA and the taxonomic studies that revealed that AAB were the most abundant in the Chilean kefir beverages microbiotas. Although genes involved in butyrate production were detected through metagenomic analyses, the genetic biosynthetic route was not complete, explaining why no butyric acid was detected. The differences in the acetic acid content between K02 and K03, where K02 showed a higher concentration of this SCFA, could be directly related to the higher relative abundance of microorganisms from the genus *Acetobacter* in its microbiota (93.43% vs. 80.99%) (Figure 1A,B). *Acetobacter* are acetic acid producing bacteria [90], and a higher proportion of these microorganisms in kefir beverages is associated with higher acetic acid content in fermented milks [4].

The microorganisms present in kefir and kefir grains form a symbiotic microbial community, where co-metabolic networks operate and allow microbial growth and, eventually, the production of this probiotic food. Metagenomic and functional analyses indicated that bacteria from the Firmicutes phylum metabolized lactose, producing glucose and galactose. Glucose is used by Firmicutes for the biosynthesis of L-lactate and D-lactate through fermentation, and yeasts can use glucose to produce acetic and propionic acid and alcohol [91]. According to metagenomic analyses, ethanol produced by yeast can be metabolized by Alphaproteobacteria to produce acetic acid by oxidation of ethanol [92]. In kefir, SCFA contribute to its organoleptic and preservative properties [93,94]. Besides its relevance for carbon and energy metabolism of the microbial ecosystem of kefir, glucose, and galactose produced by Firmicutes can be used as substrates for the biosynthesis of kefiran, the main polysaccharide of the extracellular matrix of kefir, which is essential for the formation of the macroscopic structure of kefir grain and its propagation [87]. Additionally, the biosynthetic routes for b-vitamins were identified in K02 and K03. In kefir, B-vitamins produced by yeast induce *Lactobacillus* growth, contributing to the metabolic activity in this microbial consortium [74].

One of the most biologically interesting aspects of kefir is that it is a complex ecosystem where microbe–microbe, microbe–substrate, and interkingdom interactions play a key role in the stability, spread, and perpetuation of this microcosm. One of the mechanisms of microbial interaction previously identified in fermented food is the quorum sensing (QS) communication system that regulates inter- and intra-species interactions [95]. In this study, genes belonging to QS were identified through metagenomic analyses. In this scenario, further studies to confirm the presence of autoinducer molecules in the Chilean kefir beverages are needed.

Besides genes encoding autoinducer molecules of the QS system, the metagenomes of kefir beverages contained genes involved in biofilm formation and dispersal. Metagenomic analyses and phenotypic characterization of K02 and K03 identified genes involved in curli biosynthesis and the production of the microbial β-amyloid (microbial curli), respectively. Curli is an adhesion protein involved in biofilm formation produced by different microorganisms like Proteobacteria, Bacteriodetes, Chloroflexi, and Actinobacteria [96,97,98]. Regarding kefir-associated microorganisms, microbial curli has been detected in species of *Lactobacillus*, although their role in biofilm formation has not been elucidated [99]. β-amyloid proteins are widely spread in nature with diverse biological implications. In humans, β-amyloid (human curli) is related to protein folding and processing alterations. Even though this has been directly associated with the development of neurodegenerative pathologies like Alzheimer’s disease, Parkinson’s disease, and spongiform encephalopathies [100], recent studies focused on β-amyloid-blocking agents showed that the neurodegenerative effect of these proteins is not only explained by its concentration in the nervous system [101,102,103]. On the other hand, human curli has also been associated with beneficial effects such as antiviral and antibacterial activity and the stimulation of the host defenses [104,105,106]. In bacteria, β-amyloid proteins (microbial curli) play physiological roles in adhesion, biofilm formation, and microbial resistance to the immune system and to toxins [107]. At the intestinal level, microbial curli fibers have been shown to promote intestinal barrier integrity and reduce inflammation by activating of Toll-like receptor 2 (TLR2), stimulating intestinal homeostasis [108,109]. These differences in the effect of β-amyloid fibers might be directly related to the port of entry because systemic exposure to these proteins has been shown to have detrimental systemic and nervous effects [110,111], while oral exposure promotes intestinal health, as mentioned above. In this study, microbial curli was detected in both kefir beverages (K02 and K03) by two different methods (Congo Red and curcumin staining). Considering that the production process of kefir beverages generates a ready-to-drink product, the presence of microbial curli represents an advantage due to its effect on the gut barrier and the potential application of these proteins for the treatment of gastrointestinal pathologies associated with increased intestinal barrier permeability. In this context, this field is of great interest for further studies. Moreover, it is important to highlight that this work is the first to report the presence of microbial curli in a kefir beverage.

Regarding pathogen control by this dairy beverage, genes related to biofilm dispersal were identified in this work. K02 and K03 showed an inhibitory effect on biofilm formation of *Pseudomonas aeruginosa* PAO1. In *Cobetia marina*, an inhibitory effect was observed for all the evaluated samples, except K02. Biofilm formation is a virulence factor of a wide range of pathogenic bacteria, including *Klebsiella pneumoniae*, *Escherichia coli*, and *P. aeruginosa* [112,113], contributing to host colonization and antibiotic resistance [114]. Previous reports have shown the ability of species of *Lactobacillus* to reduce bacterial biofilm formation [115,116]. Despite that K02 and K03 differed in the relative abundance of LAB (5.72% in K02 vs. 16.75%), their effect on biofilm formation did not present significant differences between both products, suggesting that this feature does not only depend on one microbial component of the kefir microbiota. On the other hand, the inhibitory effect of kefir whey was not affected by thermal treatment (95 °C), which suggests that the inhibition of biofilm formation is not enzymatically mediated.

## 5. Conclusions

The characterization of two Chilean kefir beverages, K02 and K03, revealed that these dairy products contain live and potentially probiotic bacteria, with the dominance of *Acetobacter* genus, representing a difference with other kefir beverages from different geographical origins. By their microbial structure and function, these beverages contain enzymes and biomolecules, including short-chain fatty acids, microbial curli, and inhibitors of biofilm formation, with potential biotechnological applications and health benefits. Despite their differences in the relative abundance of *Acetobacter* and *Lactobacillus* of these beverages’ microbiota, significant differences were only observed in acetic acid content, suggesting that the functional features of this type of food associated the microbial community. Finally, this is the first report of the presence of microbial curli in this type of food, opening a new area for exploring the beneficial effect of these beverages or microbial amyloid over the intestinal barrier function for the treatment of gastrointestinal pathologies involving intestinal dysfunction and inflammation.

## Figures and Tables

**Table 1:** Nutritional and microbiological properties of kefir beverages.

Characterization	Parameter	K02	K03
Nutritional	Lipid (% *w*/*w*)	3.41 ± 0.10 ^a^	3.15 ± 0.08 ^b^
	Protein (% *w*/*w*)	3.06 ± 0.06 ^a^	3.07 ± 0.08 ^a^
	Dietary fiber (% *w*/*w*)	1.71 ± 0.24 ^a^	1.28 ± 0.18 ^a^
	Carbohydrates (% *w*/*w*)	1.95 ± 0.07 ^a^	2.61 ± 0.16 ^b^
	Energy (kcal/100 g)	50.76 ^a^	51.07 ^a^
	Acetic acid (g/L)	2.71 ± 1.01 ^a^	1.78 ± 0.32 ^b^
	Butyric acid (g/L)	0.0 ± 0.0 ^a^	0.0 ± 0.0 ^a^
	Lactic acid (g/L)	6.96 ± 0.55 ^a^	6.45 ± 0.60 ^a^
	Propionic acid (g/L)	0.59 ± 0.20 ^a^	0.57 ± 0.15 ^a^
Microbiological	Total microorganism (CFU/mL)	3 × 10^9^ ± 3 × 10^8 a^	3 × 10^9^ ± 1 × 10^8 a^
	AAB (CFU/mL)	2 × 10^9^ ± 7 × 10^7 a^	3 × 10^9^ ± 1 × 10^8 a^
	LAB (CFU/mL)	1 × 10^8^ ± 3 × 10^7 a^	1 × 10^8^ ± 4 × 10^7 a^
	Enterobacteria	ND	ND
	Yeast (CFU/mL)	3 × 10^8^ ± 2 × 10^8 a^	2 × 10^8^ ± 6 × 10^7 a^

CFU: Colony forming units. ND: Not detected. Different letters denote statistically significant difference (*p* < 0.05).

**Table 2:** Enzymatic activity of kefir beverages and kefir whey.

Enzymatic Activity	K02	K02W	K03	K03W
Beta-galactosidase	+	−	+	−
Arginine dehydrolase	−	−	−	−
Lysine decarboxylase	−	−	−	−
Ornithine decarboxylase	−	−	−	−
Citrate utilization	−	−	−	−
H2S production	−	−	−	−
Urea hydrolysis	−	−	−	−
Deaminase	−	−	−	−
Indole production	−	−	−	−
Acetoin production	+	+	+	+
Gelatinase	−	−	−	−
Fermentation/Oxidation of glucose	+	+	+	+
Fermentation/Oxidation of mannitol	−	+	−	+
Fermentation/Oxidation of inositol	−	+	−	+
Fermentation/Oxidation of sorbitol	−	+	−	+
Fermentation/Oxidation of rhamnose	−	+	−	+
Fermentation/Oxidation of sucrose	−	+	−	+
Fermentation/Oxidation of melibiose	−	+	−	+
Fermentation/Oxidation of amygdalin	−	+	−	+
Fermentation/Oxidation of arabinose	−	+	−	+

## Data Availability

Sequence data reported in this study have been submitted to the National Center for Biotechnology (BioProject PRJNA388572). K02 and K03 metagenomes were submitted as BioSamples under accession number SAMN09389955 and SAMN08943003, respectively. Raw sequence data of shotgun metagenomic sequencing were deposited under accession SRR8282406 (K02) and SRR7287342 (K03). Raw sequencing data of amplicon-based 16S rDNA sequencing was deposited under the following accession numbers: SRR10232092 (K02) and SRR10233296 (K03).

## Supplementary Materials

The following are available online at https://www.mdpi.com/article/10.3390/foods11070900/s1, Figure S1: Kefir grains. Figure S2: Subsystem analysis of kefir beverages. Figure S3: Metabolism of proteins in kefir beverages. Figure S4: Metabolism of carbohydrates in kefir beverages. Figure S5: Virulence, Disease and Defense genes in kefir beverages, Figure S6: Cellulose in kefir beverages. Table S1: Composition of non-commercial culture media. Table S2: Sequence statistics for metagenomic sequencing of 16S rDNA. Table S3: Sequence statistics for shotgun metagenomic sequencing kefir beverages.
