# Supplementary material for: Metagenomic and Functional Characterization of Two Chilean Kefir Beverages Reveals a Dairy Beverage Containing Active Enzymes, Short-Chain Fatty Acids, Microbial β-Amyloids, and Bio-Film Inhibitors
Source: Foods. 2022 Mar 22;11(7):900. doi: 10.3390/foods11070900 (PMC8997647; doi:10.3390/foods11070900)
Supplement: Supplementary file 1 [file foods-11-00900-s001.zip › foods-1624582-supplementary.pdf]

Supplementary Information

Metagenomic and Functional Characterization of Two Chilean Kefir Beverages Reveals a Dairy Beverage Containing Active Enzymes, Short-Chain Fatty Acids, Microbial ß-Amyloids, and Bio-Film Inhibitors

Claudia Ibacache-Quiroga [1,2,*], Karoll González-Pizarro[2], Mariam Charifeh[2], Christian Canales[3], Rodrigo Díaz-Viciedo[2,4] and M. Alejandro Dinamarca[2,*]

Supplementary Figures

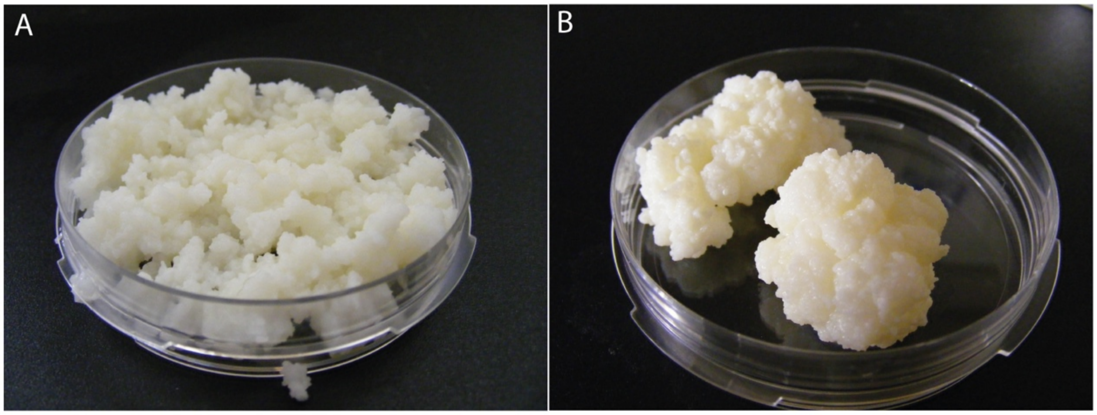

Supplementary Figure S1. Kefir grains. Kefir grains used as starters to produce K02 (A) and K03 (B) kefir beverages.

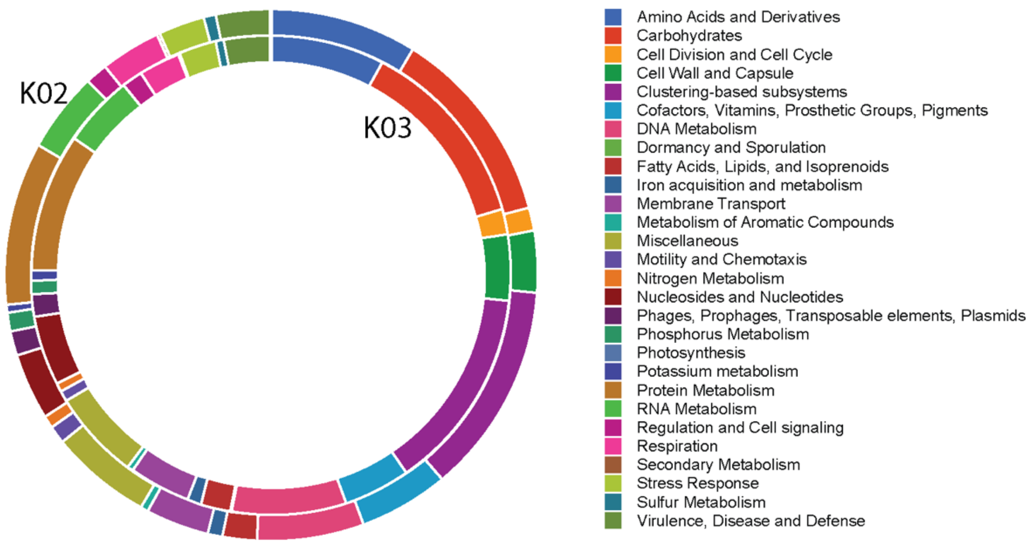

Supplementary Figure S2. Subsystem analysis of kefir beverages. Subsystem analysis of the metagenomes of kefir beverages K02 and K03, performed with MG-RAST software (v. 4.0.3).

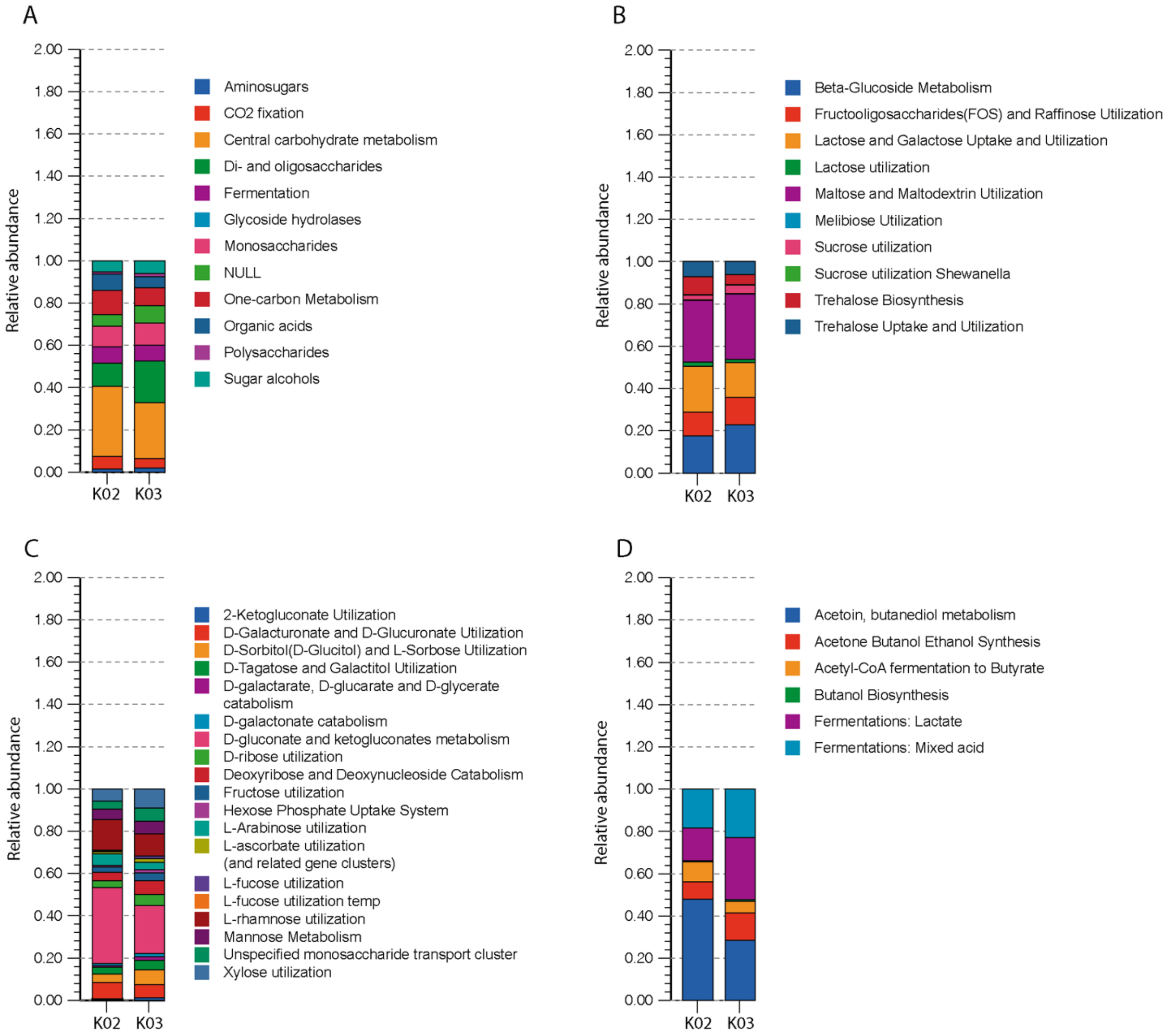

Supplementary Figure S3. Metabolism of Carbohydrates in kefir beverages. Analysis of relative abundance of genes belonging to the Carbohydrate Subsystem (A), and genes belonging to the Disaccharides and oligosaccharides (B), Monosaccharides (C) and Fermentation (D) groups. Subsystem analysis was performed using MG-RAST software (v. 4.0.3).

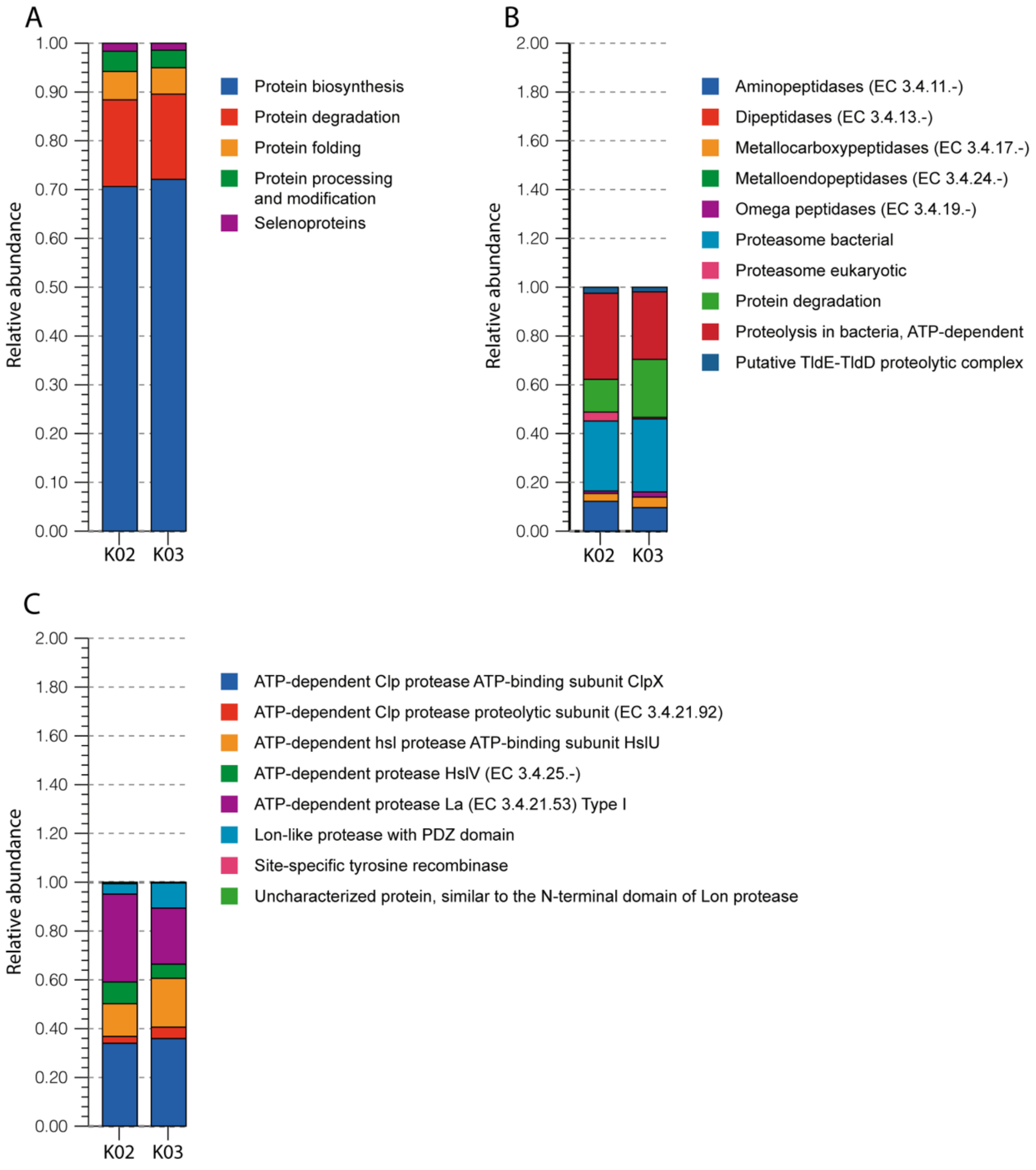

Supplementary Figure S4. Metabolism of proteins in kefir beverages. Analysis of relative abundance of genes belonging to the Protein Metabolism Subsystem (A), and genes belonging to the protein degradation (B) and Proteasome bacterial (C) groups. Subsystem analysis was performed using MG-RAST software (v. 4.0.3).

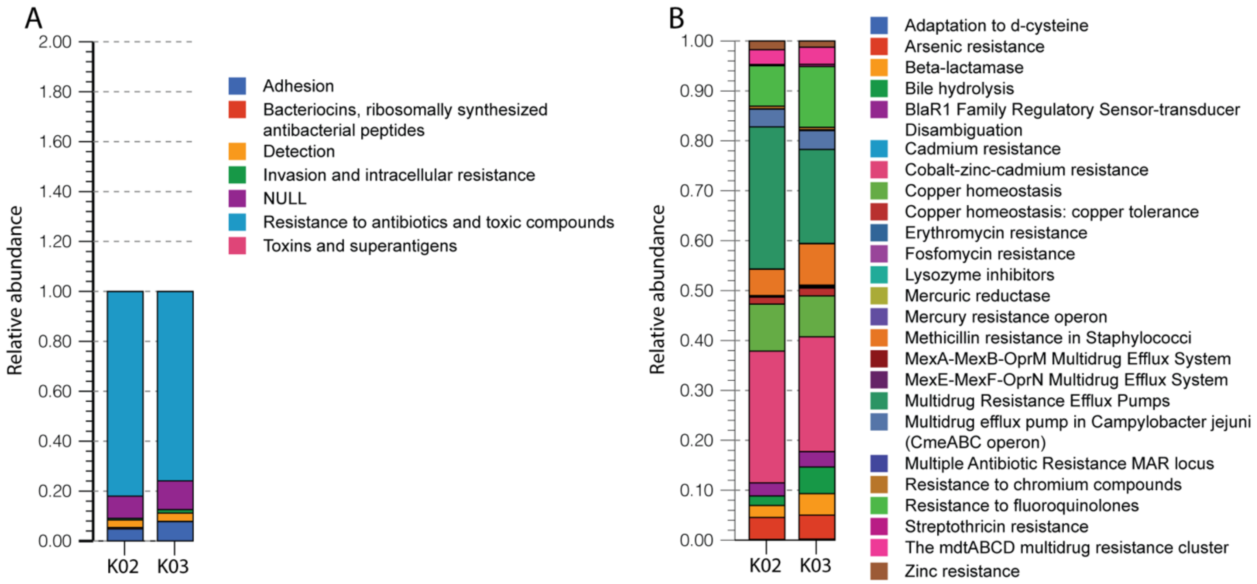

Supplementary Figure S5. Virulence, Disease and Defense genes in kefir beverages. Analysis of relative abundance of genes belonging to the Virulence, Disease and Defense Subsystem (A), and genes belonging to the Resistance to antibiotics and toxic compounds group (B). Subsystem analysis was performed using MG-RAST software (v. 4.0.3)

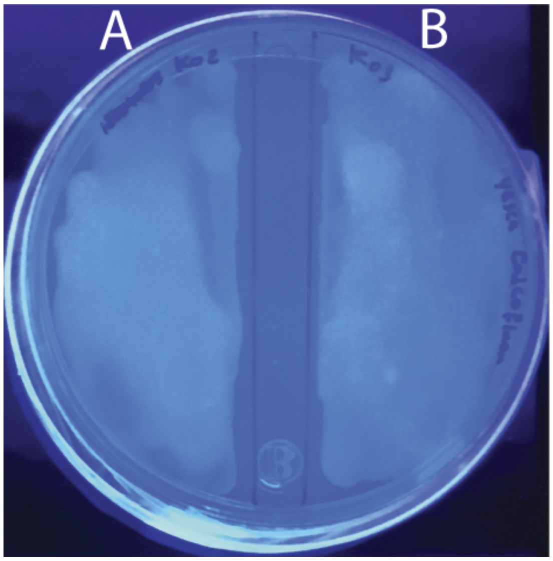

Supplementary Figure S6. Cellulose in Kefir beverages. Detection of cellulose in kefir beverages K02 (A) and K03 (B) on YESCA agar supplemented with calcofluor at 1µg/ml.

Supplementary Tables

Supplementary Table S1. Composition of non-commercial culture media

Culture medium	Composition
Glucose Yeast Calcium Carbonate Agar (GYC)	10 g/l yeast extract<br>50 g/l D-glucose<br>5 g/l calcium carbonate<br>15 g/l agar
Skim milk agar	5 g/l casaminoacids<br>2.5 g/l yeast extract<br>1 g/l D-glucose,<br>1 g/l skim milk<br>15g/l agar
LB Broth	5 g/l yeast extract<br>5 g/l NaCl<br>10 g/l tryptone
YESCA agar	10 g/l casaminoacids<br>1 g/l yeast extract<br>20 g/l agar

Supplementary Table S2. Sequence statistics for metagenomic sequencing of 16S rDNA

Sample		bp Count	Number of reads	Mean sequence length (bp)	Mean GC content (%)	Nº of OTUs
K02	Raw	141,172,792	855,933	243 ± 1	54	-
	Post-QC	395,014	194,184	243 ± 0	53	270
K03	Raw	140,783,099	854,215	243 ± 1	53	-
	Post-QC	459,164	252,960	243 ± 0	52	278

Supplementary Table S3. Sequence statistics for shotgun metagenomic sequencing kefir beverages

Sample		bp count	Number of reads	Mean read length (bp)	Mean GC content (%)
K02	Raw	13,284,079,439	131,525,539	101	54
	Post-QC		130,835,318	96	54
K03	Raw	13,368,977,211	132,266,111	101	50
	Post-QC		131,248,578	96	51
